# Preclinical Childhood Sarcoma Models: Drug Efficacy Biomarker Identification and Validation

**DOI:** 10.3389/fonc.2015.00193

**Published:** 2015-08-26

**Authors:** Brian Geier, Dias Kurmashev, Raushan T. Kurmasheva, Peter J. Houghton

**Affiliations:** ^1^Center for Childhood Cancer and Blood Diseases, Nationwide Children’s Hospital, Columbus, OH, USA; ^2^Greehey Children’s Cancer Research Institute, University of Texas Health Science Center at San Antonio, San Antonio, TX, USA

**Keywords:** human tumor xenografts, drug sensitivity, expression profiling, copy number variation, preclinical pharmacology, bioinformatics, biomarkers, drug efficacy

## Abstract

Over the past 35 years, cure rates for pediatric cancers have increased dramatically. However, it is clear that further dose intensification using cytotoxic agents or radiation therapy is not possible without enhancing morbidity and long-term effects. Consequently, novel, less genotoxic, agents are being sought to complement existing treatments. Here, we discuss preclinical human tumor xenograft models of pediatric cancers that may be used practically to identify novel agents for soft tissue and bone sarcomas, and “omics” approaches to identifying biomarkers that may identify sensitive and resistant tumors to these agents.

## Drug Development for Pediatric Cancer

Over the past 35 years, cure rates for children with hematologic and solid tumors have risen ­dramatically. For acute lymphoblastic leukemia the 5-year event-free survival (EFS) is 85–90%, whereas one-half to two-thirds of children with Ewing Sarcoma, rhabdomyosarcoma, or osteosarcoma (OS) are surviving disease-free for prolonged periods after aggressive treatment with surgery, radiation, and multiagent chemotherapy. For the remaining patients, it has been possible to slow ­progression of disease with use of intensified therapy, but cure has remained elusive. Furthermore, dose intensification/compression and introduction of new agents continues to decrease cancer mortality in children (1), although the limits of cytotoxic therapy may be close to maximal. More problematic is that these therapeutic modalities are associated with significant mortality and often long-term debilitating sequellae (2). The overriding problem is treatment failure due to the development of drug resistance. Whether this results from selection of a pre-existing clone, or through therapy-induced mutation remains to be extensively explored. A second major problem is the limited repertoire of active antineoplastic agents, targeted for childhood cancers, making it difficult to develop effective therapy for resistant tumor subtypes, even when they are identified early in the clinical course. As with recent advances in the management of adult cancers, the development of novel therapies for childhood solid tumors will require a more complete understanding of the biologic characteristics that confer the malignant phenotype that can be used to guide the integration of cytotoxic and molecularly targeted therapies most likely to confer clinical benefit.

Developing new therapies for childhood solid tumors presents certain constraints that are seldom encountered with the neoplastic diseases of adults. Childhood tumors are rare; hence, the numbers of children with a particular diagnosis restrict large-scale drug evaluation or randomized clinical trials. For example, relatively few agents receive testing in children, and from 1980 to 2003 only a single agent (teniposide) was labeled for use in children compared to more than 50 anti-cancer agents approved for use in adult oncology; furthermore, <15% of anti-cancer drugs approved for use in adult indications have labeling for children (3). As most drug-screening strategies focus on the selection of new anti-cancer agents with specific activity against adult neoplastic diseases (e.g., colon, lung, breast, etc.), agents with specific activity against childhood malignancies might not be identified.

A further restriction on drug development is that many “common” cancers of childhood respond to drugs of established efficacy, resulting in cure of a substantial number of patients. This ethically precludes the use of “experimental” agents at diagnosis. However, over the last decade, survival rates for patients with disseminated tumors at diagnosis have improved only slightly, if at all. This lack of progress is attributed, in part, to the slow rate at which most novel anti-cancer agents enter the clinical setting and the failure to optimally integrate laboratory and clinical efforts in a manner most likely to generate new therapeutic approaches with a high probability of success.

As heavily pretreated patients are most often the population recruited for Phase II trials, failure to identify a potentially useful agent could result from assessment against multi-drug resistant tumors. Thus, as we have demonstrated, an agent that shows marginal or no activity against recurrent tumors resistant to one or more drugs may have clear efficacy in advanced but previously untreated disease (4). Model systems by which such agents, or combinations of agents, can be identified, and their use optimized, are presented in this chapter. These models offer a unique resource for the development of new therapies for pediatric cancers, and offer the potential to identify biomarkers that may at some point allow patient stratification.

## Tumor Xenograft Models

### Selecting models based upon gene expression

To address some of the issues mentioned above, the NCI funded the Pediatric Preclinical Testing Program (PPTP), a consortium of groups with pediatric preclinical cancer models that could screen potential new agents and drug combinations (5, 6). Selection of suitable models for the PPTP screen involved solicitation of pediatric xenograft and cell line models from laboratories in the U.S and elsewhere. Initial screening, using cDNA array technology (7), compared 95 models with 112 patient samples representing similar histologies. Tumor models that most closely clustered with the patient samples representing the same histology were selected. A second screen (Affymetrix U133 plus 2 arrays. CEL files available at: http://gccri.uthscsa.edu/pptp) further refined the models that were included in the final screening program (8). Sixty models representing most solid tumors and acute lymphoblastic leukemia were selected for primary and secondary screens. Of these 72% are from direct patient tumor transplants into mice (patient-derived xenografts, PDX), and 48% are from tumors at diagnosis. Twenty-seven cell lines were also characterized, and demographic data for all models are available at http://gccri.uthscsa.edu/pptp.

### Fidelity of DNA copy number aberrations

Single nucleotide polymorphism (SNP) analysis demonstrated similar gains and losses of DNA copy number in model tumors as reported for the respective histotype (8), and revealed non-random events that also were highly correlated with tumor type (8). All models were DNA fingerprinted using short tandem repeat (STR) assays, and profiles filed as a reference for determining fidelity of lines during passage. More recently, each model has been characterized using the Agilent’s SurePrint G3 Gene Expression microarray platform where four replicate tumors approximately 200–300 mm^3^ per tumor line were used to create a more robust expression profile dataset. Exome sequencing has been completed for approximately 90 cell line and xenograft models. Thus, it is now possible to test the sensitivity of a particular model based upon an “actionable” mutation (9, 10).

### Long non-coding RNAs

The Agilent Sureprint G3 Gene Expression version 1 array is able to measure 34,809 unique mRNA variables, which is far more than previous Affymetrix platforms that currently dominate the vast collection of arrays found in the Gene Expression Omnibus (GEO). A novel feature of this particular array is the measurement of long-intergenic non-coding RNAs (lincRNA). The lincRNAs provide an additional transcriptomic perspective that is valuable in understanding tumor biology (11) and may explain variation in response to drug treatment. In our analysis of pediatric solid tumors, we observed that lincRNA expression is able to discriminate cancer populations as accurately as protein coding gene expression. Such an observation is interesting and points to the relevance of lincRNA in studying malignant disease. Notwithstanding this interesting yet isolated molecular view, the real power of cancer genomic data lies in the ability to integrate different levels of molecular evidence to elucidate novel insights about cancer biology (12, 13).

## Establishing an *In Vivo* Screen

### Response criteria

**Figure 1:**
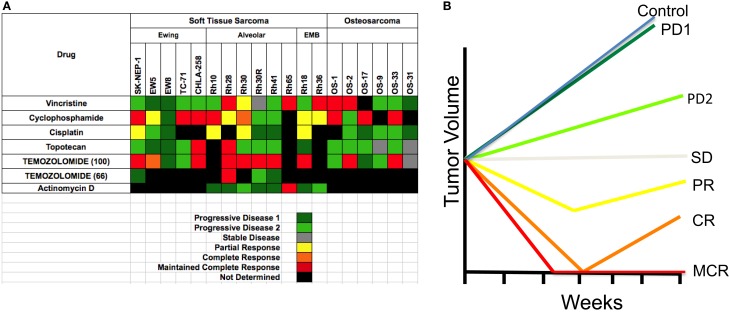
**(A)** Heat map representation of the standard cytotoxic drugs screened by the PPT. Xenograft tumor models are shown at the top, grouped by histotype. Agents tested are shown in the left column. **(B)** The graph shows a representation of tumor responses, and the designation of the response.

One of the reasons that preclinical models have generally failed to predict clinical utility of agents is the different criteria for assessing activity in the model compared to the clinic. For example, inhibition of tumor growth rate by 80% in the laboratory is regarded as biologically significant, whereas a similar effect in a patient is classified as progressive disease. For the PPTP screen, response criteria were “modeled” after clinical response criteria, and that an active agent should cause objective tumor regression. These criteria were based upon several preclinical studies that related regressions in mice to responses of agents in phase I clinical trials. Notably regression of rhabdomyosarcoma xenografts to melphalan, topotecan, irinotecan, and camptothecin combinations (14–17), as well as neuroblastoma xenografts (16, 18), correlated with activity in clinical trials (4, 19–22). Using these criteria to define activity, known clinically effective agents could be identified. Similarly, criteria for acute lymphoblastic leukemia models were developed that identify known clinically identified active agents (23). Preclinical models of medulloblastoma accurately predicted the clinical activity of topotecan (24). Models of Wilms tumor (nephroblastoma) also identified known active drugs (cyclophosphamide, vincristine) using these criteria as did Ewing sarcoma models (cyclophosphamide, cisplatin). Validation of other models is ongoing through a series of clinical trials being conducted through the Children’s Oncology Group (COG). The PPTP developed response criteria that resemble clinical response criteria, fully recognizing that both cytostatic as well as cytotoxic agents would be evaluated (6). Each tumor within a treatment group is given a score dependent on the response [progressive disease 1 (PD1)] where there is <50% growth inhibition scores 0, whereas maintained complete response (25) scores 10. The group score is the median. This allows large datasets to be reduced to a “Heat Map” format, as shown in Figure 1A for standard cytotoxic agents screened against sarcoma models. The heat map format allows comparison of multiple drugs and shows that the objective response rate (ORR) for “known” actives (vincristine, cyclophosphamide, cisplatin, and topotecan) is approximately 40%. Figure 1B shows a schematic of the median tumor response for each response classification.

## Evaluation of Standard Cytotoxic Agents

All solid tumor testing to date in the PPTP used subcutaneous models, whereas for acute lymphoblastic leukemias (ALL) disseminated models were used. This review will focus only on the responses of sarcomas. One way to validate preclinical models (“model” is defined as a panel of tumors having the same pathologic diagnosis) is to ascertain whether the model identifies agents of known utility against the disease in children. Standard agents such as vincristine showed activity (i.e., induced tumor regressions ≥50%) in RMS models but no activity against EWS xenografts. Cyclophosphamide showed activity in all three tumor types, whereas cisplatin was active in some EWS and RMS models. Topotecan also demonstrated activity against EWS and RMS models, with disease stabilization in two OS models. Thus, the models identify agents with known single agent activity in these pathologies. Overall, sarcoma models showed marked sensitivity to anti-mitotic agents with an ORR of 34.7% when tested in mice at the maximum tolerated dose/schedule (MTD). Temozolomide, used in combination treatment of relapse sarcoma, showed broad-spectrum activity when tested at the MTD in mice. By contrast, a dose level in mice giving systemic exposure on the high side of that achievable in humans (66 mg/kg, Figure 1) showed activity only against Rh28 RMS that is deficient in MGMT required for repair of O^6^G adducts (26, 27).

**Figure 2:**
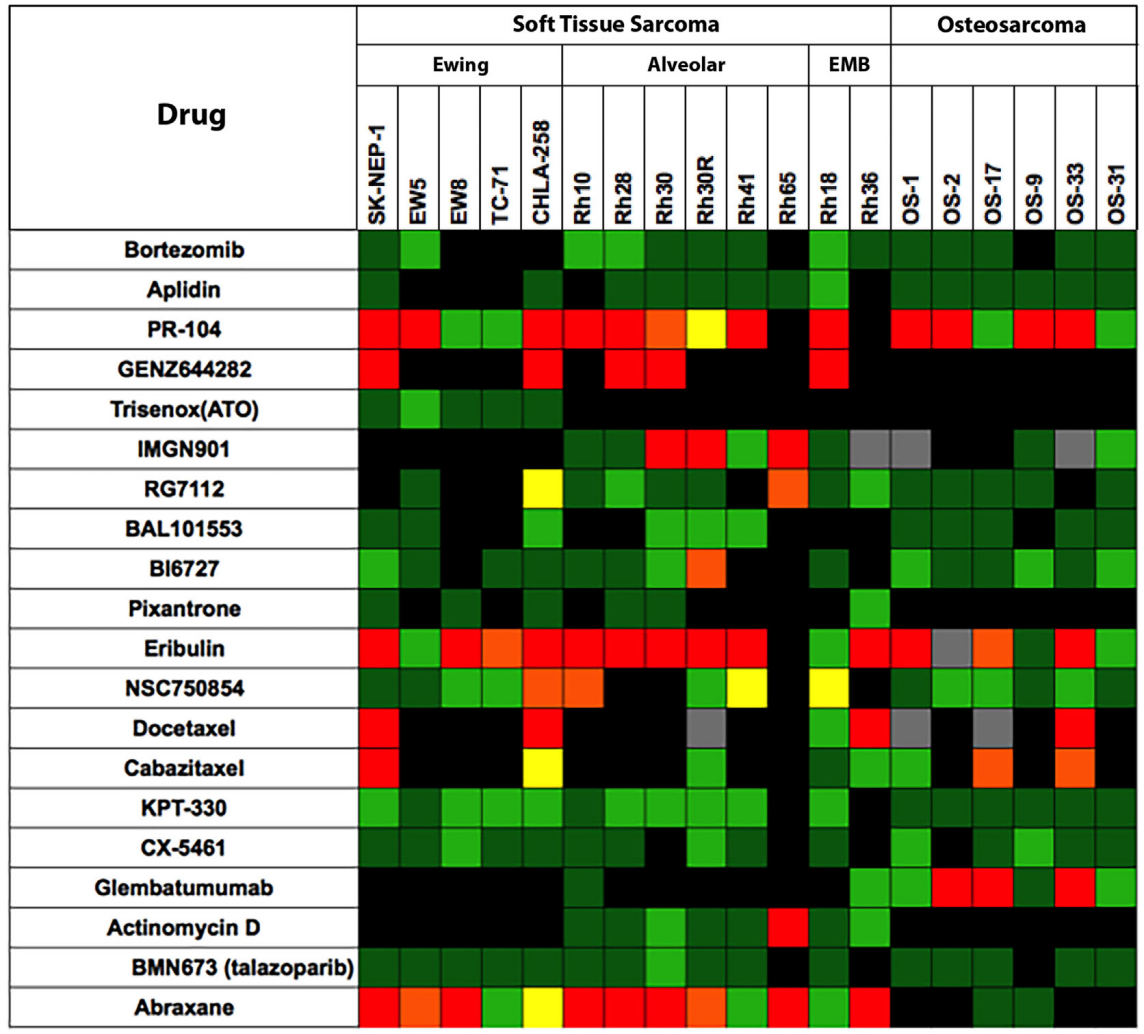
**Efficacy testing results for 20 cytotoxic agents tested against sarcoma models by the PPTP**. Color codes are as for Figure 1.

The testing of experimental cytotoxic drugs against the OS, EWS, and RMS panels is presented in Figure 2 in “Heat Map” format (6). For eribulin (28) and abraxane (29), plasma exposures to these drugs in mice, at the doses tested, appear relevant for human exposure, whereas exposures to docetaxel and cabazitaxel substantially exceed those attainable in humans. As shown above, the models are responsive to anti-mitotic agents, perhaps reflecting a high proliferative fraction in xenograft models. By contrast, the tubulin-binding agent, BAL101553, showed no significant antitumor activity against sarcoma models. Hence, tumor sensitivity is not necessarily a consequence of increased proliferation.

The alkylating agent PR-104, a pre- pro-drug activated under hypoxia and by the aldoketo reductase AKR3C3 (30, 31), showed significant broad-spectrum activity when tested at the mouse maximum tolerated dose/schedule (MTD). However, at dose levels in mice that approximate human drug exposure, PR-104 was not active against solid tumor xenograft models. The non-camptothecin topoisomerase I inhibitor, GENZ644282, was active against SK-NEP-1 Ewing sarcoma, whereas topotecan was not. Other cytotoxic agents having novel mechanisms of action [aplidin, KPT-330 (selinexor, a CRM1/XPO1 inhibitor), CX-5461 (RNA pol I inhibitor)] and the PARP1 inhibitor, BMN-673, showed little or no antitumor activity against sarcoma models.

## Evaluation of Signaling Inhibitors

**Figure 3:**
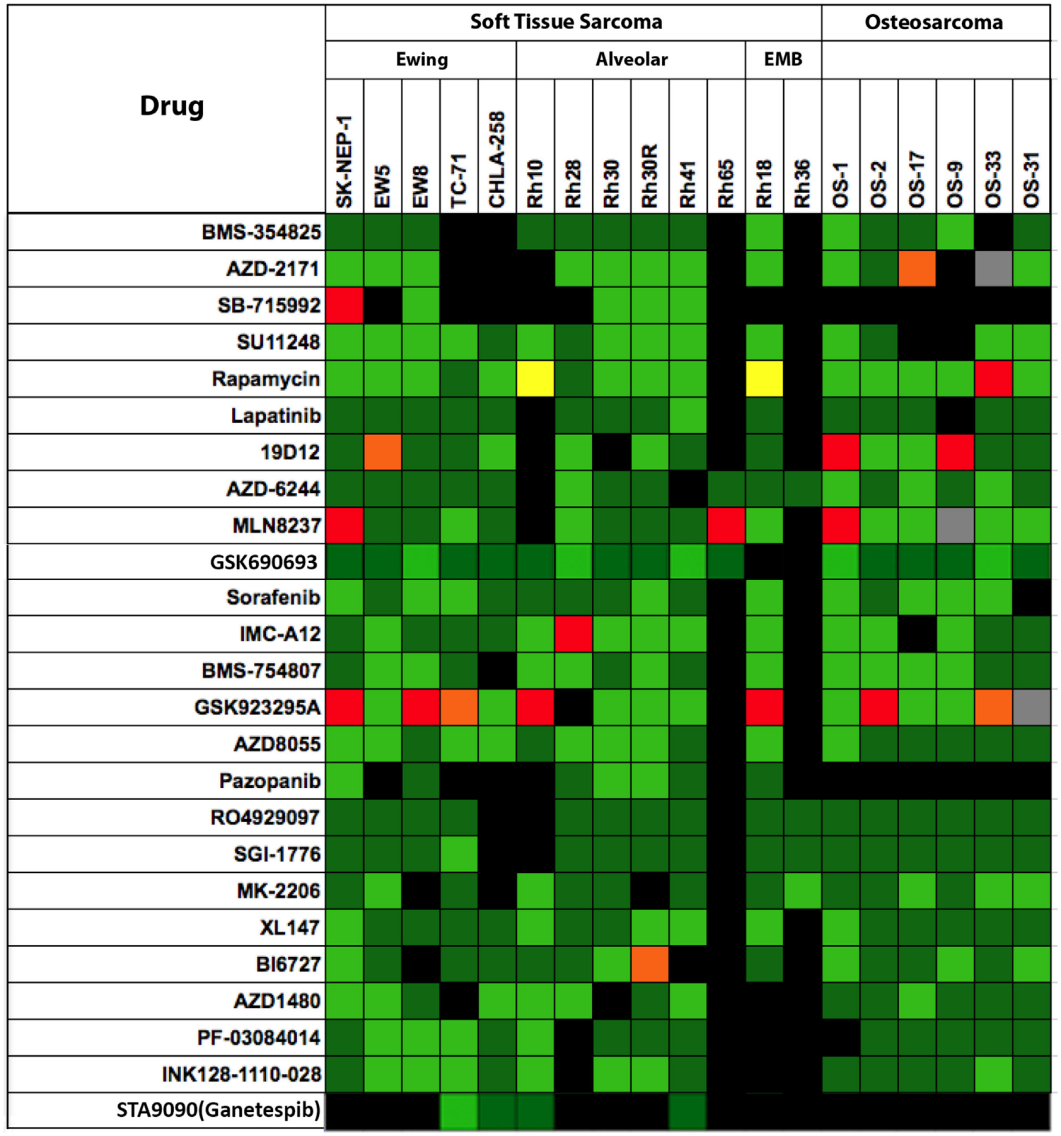
**Efficacy testing results for 25 signaling inhibitors tested against sarcoma models by the PPTP**. Color codes are as for Figure 1.

Shown in Figure 3 are testing results for 25 “signaling” inhibitors. These include classical inhibitors of the IGF–PI3K–TOR pathway including antibodies and drugs targeting IGF-1R (19D12, IMC-A12, BMS-754807), and small molecule drugs that selectively inhibit PI3K (XL-147), AKT (MK-2206), TOR (rapamycin, AZD8055, INK128), MEK (AZD6244) as well as multikinase inhibitors (sorafenib, SU11248, cabozantinib), and inhibitors of mitotic kinases (MLN8237, BI6727). In this dataset, there are 357 tumor/drug evaluations. The ORR was 5.6% (20/357 tests). Of these, inhibitors of mitotic kinases [PLK1 (BI6727), Aurora kinase (MLN8237)], and the kinesin inhibitor (GSK923295A) showed the greatest activity, consistent with the activity of other “non-signaling” anti-mitotic drugs (vincristine, eribulin). Excluding the responses to mitotic inhibitors in the “signaling” drug set, the ORR was a dismal 2.4% (9/291 tests).

## Critical Evaluation of PPTP Models

The PPTP used exclusively xenograft models, hence these preclinical studies are useful for identifying agents that work predominantly via direct action on tumor cells. Xenograft models are, by definition, not suitable for evaluating immune-regulators, and the stromal elements are mouse. Despite these obvious limitations, these sarcoma xenografts identify each of the cytotoxic drugs known to be active, and have identified novel agents and combinations that have advanced to clinical evaluation through COG. The ORR to signaling inhibitors is disappointingly low (2.4%), which is of concern. However, there is reason to consider that these results are going to be representative of the clinical activity of signaling agents when given individually. For example, notable exceptions are the response to selumetinib (MEK inhibitor) in an astrocytoma with a BRAF^V600E^ mutation (9, 32), the complete response to dasatinib in the Ph^+^ ALL-4 xenograft (33), expected based on the preclinical and clinical activity for dasatinib against Bcr-Abl expressing leukemias and responses in Ewing sarcoma and other sarcomas to IGF-1 receptor targeting antibodies (34, 35). Although PPTP did not test crizotinib, ALK-mutant or ALK-amplified neuroblastoma xenografts included in the PPTP neuroblastoma panel were responsive to this agent (36, 37). These results suggest that subcutaneous xenografts can indeed identify both cytotoxic drugs and signaling inhibitors that have clinical utility against the appropriate cancers in children, and hence are an appropriate primary screening tool. However, if these preclinical results are relevant to clinical responses, it is clear that developing agents of this class will yield a very low response rate, and that matching inhibitor to patient tumor characteristics will be required.

From the PPTP experience, the major factor that prevents accurate translation of preclinical data to the clinic is the difference in drug exposures in mice compared to those achieved in children (38). If differential host tolerance is normalized, then the predictive value of the preclinical data appears to be good. Obviously, there will be exceptions. For example, drug access to brain may limit the use of a drug shown to be effective against brain tumors when grown subcutaneously in mice. However, secondary orthotopic models can relatively easily identify these “false positive” results.

Another issue is the site of growth – heterotopic (subcutaneous) or orthotopic? Clearly, the subcutaneous sarcoma models identify known active agents, and accurately predict for clinical activity (melphalan, camptothecins, etc.), thus fulfill the basic function as set out by the PPTP. Whether drug activity dramatically differs in orthotopic models requires rigorous experimentation, and use of endpoints that can be equated between subcutaneous and orthotopic models. One problem in comparing heterotopic and orthotopic models is that tumor volume at the start of therapy is often significantly smaller in orthotopic models, hence these tend to be more sensitive by virtue of size (drug access?).

## Mining for Biomarkers of Drug Response

### Expression profiling

As noted by the NCI-EORTC Working Group on Cancer Diagnostics, the number of markers that have emerged as clinically useful is very small. One of the problems has been small datasets, and initial promising results have not been validated in larger trials (39, 40). Fully realizing the limitation of relatively few preclinical models (~50) and within a tumor type very few models (5–10), thus, at best, our correlations derived from expression data and response data are hypothesis generating. Gene expression profiles have been established for both cell line panels and the xenograft models, as well as SNP profiles. Thus, potentially, sensitivity *in vitro* can be correlated with either expression patterns or DNA copy number variation (CNV). Such profiles could then be tested for predictive value for response against the *in vivo* cancer models. Alternatively, expression or CNV profiles that correlate with sensitivity or resistance to an agent in the animal models may predict those patients who may benefit from this treatment. However, although data may be obtained on almost 50 models, it is best to consider, at this time, such data as hypothesis generating. For the analyses presented, we have used data from all models, and not just from soft tissue and bony sarcomas, as there are too few models for which data are available.

As was illustrated by Lander, the greatest challenge to revealing the fruit of nature by omic technology is in our ability to succinctly probe and dissect millions of read outs within the global scope of a sparse random realization (22). In general, the dimensionality digestion of genome-wide mRNA is complex in two-sample experiments and becomes even more so when considering large cohorts of diverse samples. For example, in preclinical drug evaluation, the biological diversity of samples within sensitive or resistant xenografts is likely heterogeneous and not sampled uniformly.

(1.1) $$\theta \left(x,\;y\right)=\frac{\mu \left(x\right)-\mu \left(y\right)}{\sigma \left(x\right)+\sigma \left(y\right)}$$

(1.2) $$y=\beta_{0}+\sum_{i=1}^{p}x_{i}\beta_{i}+\in$$

(1.3) $$E\left(y\right)={\hat{\beta }}_{0}+\sum_{i=1}^{p}{\hat{\beta }}_{i}x_{i}$$

### Equation set 1: Measures of mRNA association

Classically, the mRNA difference between two classes is evaluated by the so-called signal-to-noise or simply stated as the difference between means relative to the variances, see equation 1.1, where *x* is the log distribution of class A mRNA and *y* is the log distribution of class B mRNA, respectively. In this scenario, two classes are statistically different if the mean separation is large relative to the variance within each class, which is typically assessed by permuting the class labels several times to estimate an empirical probability of observing the realized statistic (24) or more elegantly by bootstrapping if sample size permits. Such an approach is useful when considering treatment-condition effects or lineage differences in biological experiments. However, when considering a diverse set of tumors whose preclinical drug outcome does not necessarily follow lineage trends, there is a lack of statistical difference between classes after compensating for multiple hypothesis testing. Additionally, a class label is likely not perfect to discriminate and to guide biomarker discovery unless the drug would tailor to specific cancer disease characteristics. Furthermore, on a genome-wide scale we have found the mapping between mRNA and drug sensitivity to be problematic unless a continuous random variable is considered.

In cancer cell sensitivity modeling with microarrays, the linear relationship between basal mRNA measurements and drug sensitivity is a simplistic analytical approach to generate new hypotheses about a drug’s chemical biology (41–43). From a statistical perspective, the case of linearity is argued because microarray model inputs and sensitivity outputs are typically normally distributed and those examples that do not follow a normal trend can be discarded as outliers. Whether or not our variables are specifically tied to the pharmacodynamic action is an afterthought. Rather, large-scale microarray data mining is able to identify a set of concerted changes that are associated with drug sensitivity. The dissection of the molecular pattern with regard to drug sensitivity is not possible unless additional experiments are performed; for example, RNA interference or preclinical xenograft validation. As an alternative to experimentation, the molecular pattern or “hits” discovered are queried against public databases that integrate several molecular data levels to attest whether or not the pattern is associated with, for example, survival, or a specific cancer population. Moreover, any approach in machine learning or predictive inference involves training and validation using statistically independent realizations of a given process. Cross-validation, a statistical technique to estimate prediction error, is absolutely necessary when selecting biomarkers but may still reveal poor predictors because such few samples are available or the underlying data are not representative. However, the coupling of cellular screening with preclinical xenograft studies may provide a reliable platform to identify robust biomarkers or de-prioritize the significance of cellular biomarkers. Those molecular features that are predictive in both model systems are likely indicative of sensitivity.

The dependent variable choice can vary by drug but usually involves the relative half maximal inhibitory concentration (rIC_50_) *in vitro* or relative tumor regression *in vivo*. In order to estimate linear coefficients between rIC_50_ and mRNA, we use a high-dimensional method introduced by Zou and Hastie coined the elastic net (44). The elastic net is a regression optimization that considers all probable model fits efficiently, which performs variable or model selection in a continuous rather than one-model-at-a-time discrete manner; those variables not influential in predicting y have linear coefficients, i.e., β’s, set to zero.

(2.1) $$\begin{array}{c} \min\\ \beta_{0},\beta \end{array}\left[\frac{1}{2n}\sum_{i=1}^{n}\left(y_{i}-\beta_{0}-x_{i}\beta \right)+\lambda P_{\alpha }\left(\beta \right)\right]$$

(2.2) $$P_{\alpha }\left(\beta \right)=\sum_{j=1}^{p}\left(\frac{\left(1-\alpha \right)}{1}\beta_{j}^{2}+\alpha \left|\beta_{j}\right|\right)$$

### Equation set 2: Elastic net regression

The objective function and criteria for guiding the process are shown in equation set 2 and is easily executed using a software implementation provided by the Matlab^®^ Statistics Toolbox. As a custom pre-processing step, only genes with a significant univariate correlation are considered initial inputs to the elastic net algorithm. The genes identified by univariate correlation are an associated subset of all possible genomic correlates and are dictated by an arbitrary local type I error rate that vastly underestimates the realized type I error. Whether or not we incur false positives is of no concern, as these will be removed by the elastic net regression. The α parameter, shown in equation 2.2, is able to pool several correlated features and eliminate those that are not informative. The α that results in the lowest mean squared error, based on 10-fold cross-validation, is selected as the best model and hence most predictive gene network. A critical and sometimes overlooked step in predictive model building is the correct utilization of cross-validation, as illustrated well by Hastie, Tibshirani, and Friedman (45). This includes any initial gene selection steps being in the cross-validated estimate of prediction error. In our approach, we pre-process the gene list by removing any genes that are not significantly correlated and this step is included in the cross-validated error estimate for different α values. On the other hand, our global pre-processing steps that exclude any information about our target function are performed prior to any modeling, which include z-score transformation of inputs and outputs as well as removal of training samples whose output is not consistent with a normal probability curve.

### Example, anti-mitotic drugs for biomarker application in PPTP

The drugs, MLN8237 (alisertib) (46, 47) and BI6726 (volasertib) (48), are both somewhat effective anti-mitotic targeted therapies evaluated by the PPTP that inhibit Aurora kinase A (AURKA) and Polo-like kinase 1, respectively. The cellular sensitivity of these kinase inhibitors is quite striking and showed cell growth inhibition across most pediatric cell lines screened. The drugs, eribulin and vincristine, are both highly effective agents that target microtubule dynamics in general. These two drugs were shown to be very active in the PPTP xenograft panel. Vincristine is a “known” active agent being used in many “standard-of-care” protocols, whereas eribulin has just entered phase I testing in children as a cancer therapeutic. Both drugs were potent cytotoxics *in vitro* with a median rIC_50_ concentration of 0.224 and 0.2 nM, respectively. These drugs, in the examples that follow, show a range of predictability between *in vitro* and *in vivo* systems. Additionally, we are able to hypothesize global predictors of agents that target microtubule dynamics by comparing signatures (47, 49, 50).

In these examples, we are able to show whether or not *in vitro* drug sensitivity models are valid by applying receiver-operating characteristic (ROC) curve analysis to known xenograft outcomes. For these analyses, we used a binary system dividing responses into disease progression [progress disease (PD)] or progression-free disease that included objective regression and stable disease (MCR, CR, PR, SD), and model predictions. As we noted before, the *in vitro* prediction is a continuous random variable that summarizes expected rIC_50_, *y*, given changes in mRNA, *x*. That is, a single xenograft has a composite score defined by the linear combination of mRNA features derived *in vitro*. In general, discriminatory power is defined as the trade off between sensitivity and specificity, respectively. A ROC curve measures the discriminatory power of a score when applying different score thresholds rather than measure performance at a single arbitrary cut off, i.e., positive predicted values are sensitive while negative predicted values are resistant, and is reported overall as the area under the ROC curve (AUC); for more detail, see Ref. (51).

### Vincristine

To “calibrate” the PPTP tumor panels, we evaluated the standard chemotherapeutic agent, vincristine, an agent included in the backbone of most treatment regimens for solid tumors and acute lymphoblastic leukemia. Vincristine binds to tubulin dimers, the subunits of microtubules, inhibiting assembly of microtubule structures. Disruption of the microtubules prevents formation of the mitotic spindle required to segregate chromosomes and arrests mitosis in metaphase. Although the basis for selectivity for tumor vs. normal cells is not fully understood, vincristine is a component of most curative therapies used for treatment of pediatric cancers, although the proportion of patients who benefit from vincristine may be 30–50%. Thus, identifying biomarkers for response may assist in identifying patients whose tumors would be sensitive to this drug. As shown in Figure 1, vincristine was evaluated against five Ewing sarcomas (SK-NEP-1, EW5, EW8, TC-71, and CHLA258), six alveolar rhabdomyosarcomas (Rh10, Rh28, Rh30, Rh30R, Rh41, and Rh65), two embryonal rhabdomyosarcomas (Rh18 and Rh36), and six OSs (OS-1, OS-2, OS-9, OS-17, OS-31, and OS-33). Objective regressions were observed in four rhabdomyosarcoma models and two OS models. Additional regressions were observed in Wilms tumor, and all eight ALL models (not shown).

Limited single agent data on vincristine in OS are available from the 1960s (19, 20). Several subsequent single arm and randomized trials combining vincristine with other conventional agents failed to clearly demonstrate a role for vincristine in neo-adjuvant chemotherapy. There have been few recent clinical trials of microtubule-targeted therapies in OS (reviewed in (52)). In an Italian pediatric solid tumor phase 2 study, a response to vinorelbine was observed in one of five patients with OSs (53). However, OS is not generally considered to be sensitive to anti-mitotic agents. As the proliferative fraction of xenografts is greater than that in the patient tumors, it is probable that anti-mitotic agents show as more active in the models that they are in the clinic.

The elastic net regression algorithm selected 188 mRNA variables, 35 of which were lincRNAs, based upon the log rIC_50_ of 22 PPTP cell lines. The *in vitro* linear model with these 188 mRNA inputs predicted 44 solid tumor xenograft outcomes (26 PD, 1 SD, 3 PR, 2 CR, and 12 MCR) very well with an area under the curve of 0.88. According to Ingenuity Pathway Analysis (IPA) (Ingenuity^®^ Systems, www.ingenuity.com), MAPK9, MARK2, NEFL, PVRL3, and SHC1 biomarkers are involved in microtubule dynamics. Interestingly, MARK2, a sensitive correlate, is important for microtubule stability (41) and has been shown to slow microtubule growth upon *in vitro* knockdown (54). Potentially, tumor cells that are rich with MARK2 indicate that they are more reliant on efficient microtubule dynamics to proliferate, and hence, are more reliably targeted by vincristine.

Of note, our analysis did not identify ABCB1 as a significant predictor, whereas there is an extensive literature that attests to vincristine being transported out of cells via this efflux pump. A primary caveat to our analysis approach is that we initially filter out genomic correlates at an arbitrarily chosen local type I error rate. Additionally, the linear regression approach dictates that the best predictors will be normally distributed; as this will produce the lowest mean squared error, given that a linear model is essentially predicting the expected value. In total, there were 1,604 possible genomic correlates when deriving our linear regression model. ABCB1 was not even considered because it was weakly correlated relative to other genomic correlates, and hence, did not pass our local type I error threshold. However, upon visual inspection of ABCB1 DNA copy number and mRNA across the panel of cells and xenografts tested, we see that a pattern does exist but is non-linear and ABCB1 mRNA is, in general, not normally distributed. This particular pattern is a good example of how a linear regression approach, robust as it may be, will overlook “interesting” dimensions whose activity is limited to only a subset of samples.

Vincristine is an established drug that is usually combined with actinomycin D, doxorubicin, or cyclophosphamide and has demonstrated success in pediatric cancer patients. Our signature may perhaps identify patients that have an increased likelihood of responding to vincristine treatment alone. Furthermore, the excellent validation performance and significance of discovered biomarkers prioritize this signature for additional validation and potential for clinical utilization as a companion diagnostic marker when treating with vincristine alone.

### Eribulin

**Figure 4:**
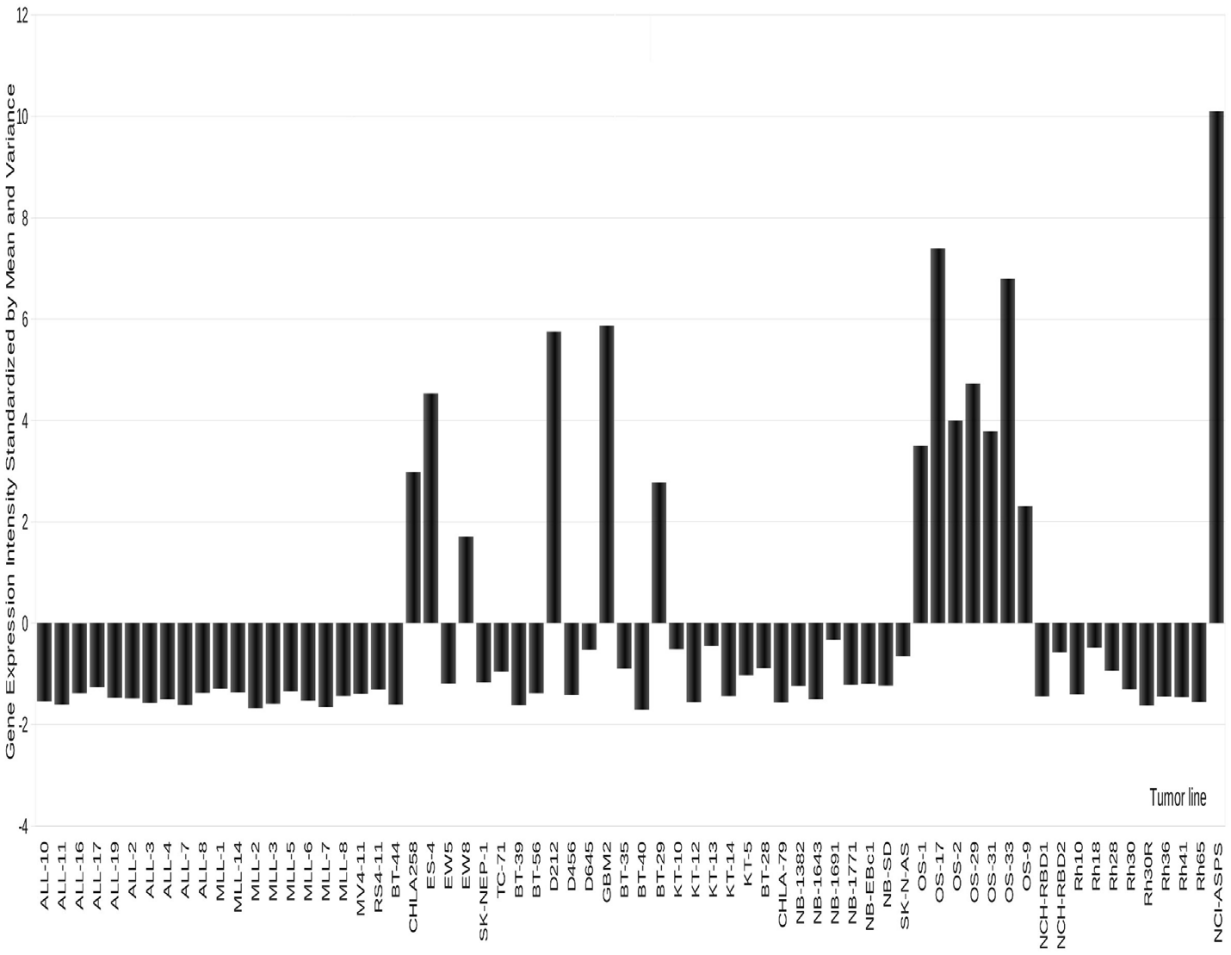
**Expression of GPMNB in PPTP xenografts**. High-level expression is detected in osteosarcoma, and some Ewing sarcoma models as well as in two glioblastoma xenografts.

Eribulin is probably the most active agent evaluated in the PPTP screen, causing tumor regressions of 18 of 35 (51%) of solid tumor models and all eight acute lymphoblastic leukemia models, Figure 4 (28). Of note, drug exposures in mice causing regressions of tumors appear similar to patient exposures reported from adult clinical trials. Eribulin is a fully synthetic macrocyclic ketone analog of halichondrin B, a natural product derived from the marine sponge Halichondria okadai (55, 56). Halichondrin B and eribulin are capable of inducing irreversible mitotic blockade and apoptosis by inhibiting microtubule dynamic instability (57). Dynamic instability applies to the growth and shortening of microtubules required for mitosis. Eribulin inhibits microtubule growth by binding with high affinity at the plus ends (58). The mechanism of inhibition of microtubule dynamic instability by eribulin is distinctive from that of other tubulin-binding anti-mitotic agents in that eribulin suppresses the growth parameters at microtubule plus ends without affecting microtubule shortening parameters (58, 59).

Analysis of the eribulin data with approximately equal numbers of responding and non-responding solid tumor xenograft models, thus provided an interesting test of the value of the “omics” database. The elastic net regression algorithm selected 139 mRNA variables, 36 of which were lincRNAs, based upon the log rIC_50_ of 22 PPTP cell lines. The *in vitro* linear model with these 139 mRNA inputs predicted 25 solid tumor xenograft outcomes (8 PD, 2 SD, 1 PR, 4 CR, and 10 MCR,) quite well with an area under the ROC curve of 0.7. According to IPA, ATXN2, BBS10, DLG4, EFNB2, KIF18A, NUSAP1, and PTPRM biomarkers are involved in microtubule dynamics. Interestingly, NUSAP1, a sensitive correlate, is reportedly involved in several cellular processes relevant to eribulin mechanism that covers segregation of sister chromatids, condensation of mitotic chromosomes, mitosis, bundling of microtubules, and aberration of mitotic spindle (60) as well as morphology of mitotic spindle (61). KIF18A, another sensitive correlate, is also quite interesting. Kinesin family member 18A is reportedly involved in alignment and congression of chromosomes (62) as well as de-polymerization of microtubules (63). Another noteworthy biomarker is ABCB1, a protein that encodes a drug transporter MDR1b (also known as P-glycoprotein). ABCB1 transports a variety of hydrophobic drugs, including eribulin (64). Furthermore, the decent validation performance and significant relevance of discovered biomarkers prioritizes this signature for additional validation and potential clinical utilization as a companion diagnostic marker in the treatment of pediatric cancer patients.

### Alisertib (MLN8237): An inhibitor of aurora kinase A (AURKA)

**Figure 5:**
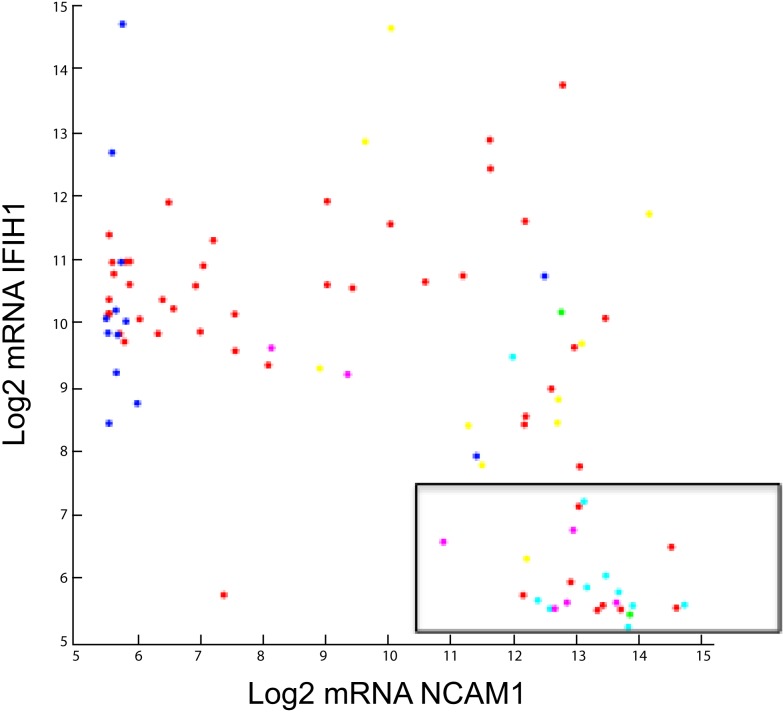
**Log2 Agilent mRNA pattern between NCAM1 and IFIH1**. Seneca Valley Virus (NTX-010) sensitivity as defined by PPTP is overlaid. The boxed area shows 24 of 26 cell lines and xenografts that were sensitive to NTX-010.

The Aurora serine/threonine protein kinases are a family of three kinases (Aurora A–C) with different tissue and temporal expression profiles. These enzymes play key roles in mitosis and meiosis, defects in which can lead to abnormal mitotic events and induction of programed cell death (apoptosis) (65). AURKA is essential, as is highlighted by the fact that genetically engineered null mice are embryonic lethal (dying at the blastocyst stage) (66). AURKA activity is also required for centrosome duplication and separation, microtubule-kinetochore attachment, spindle checkpoint, cytokinesis (67, 68), the G2/M transition (69), and phosphorylation of Polo-like kinase 1 (70). Furthermore, AURKA has been implicated as an oncogenic driver in human cancers (71). AURKA has been found to be over-expressed in cancer cells and the AURKA gene locus is amplified in selected adult tumors (72). When tested by the PPTP at the maximum tolerated dose/schedule (MTD), alisertib exhibited good activity, notably against neuroblastoma and ALL models (46), Figure 5.

Analysis of this dataset using the elastic net regression algorithm selected 69 mRNA variables, 24 of which were lincRNAs, based upon the log rIC_50_ of 22 PPTP cell lines. Despite a strong training validation, the *in vitro* linear model with these 69 mRNA inputs predicted 39 xenograft outcomes (20 PD, 4 SD, 1 PR, 4 CR, and 10 MCR) poorly with an area under the curve of 0.48 or practically random discrimination. According to IPA, there were no biomarkers that had a documented interaction with the drugs target, AURKA. Furthermore, the poor validation performance and insignificance of discovered biomarkers with respect to the molecular target de-prioritizes any additional validation or clinical utilization of this signature. In this example and given the data at-hand, the spectrum of cellular sensitivity observed is not translatable to preclinical xenograft models with respect to messenger-RNA.

### AURKA copy number

In contrast to expression profiling, gene copy number analysis for AURKA appears to support an inverse relationship between AURKA expression and sensitivity. Increased copy number was present 14 of the solid tumors. Loss of copy number was detected in seven solid tumors and one leukemia model. Furthermore, the correlation between gene expression variation and CNV was strong, placing this locus in the top 1.6% of all genes tested. While there is no absolute relationship between CNV and tumor sensitivity, of the 14 solid tumors with increased copy number, there were only two that showed sensitivity to alisertib. By contrast, five of the eight models demonstrating decreased copy number were sensitive models to alisertib (46). It is of note that at drug exposures achieved in patients, only the most sensitive preclinical models (ALL) are likely to respond to treatment. However, several rhabdoid tumor models were relatively sensitive to alisertib, and responses were observed in several patients with CNS rhabdoid tumors (73).

### Volasertib (BI6727): An inhibitor of polo-like kinase 1 (PLK1)

*In vitro* volasertib demonstrated cytotoxic activity (median rIC_50_ value of 14.1 nM, range 6.0–135 nM), and at the MTD-induced significant differences in EFS in 19 of 32 (59%) of the evaluable solid tumor xenografts and in two of four of the evaluable ALL xenografts. Objective responses (CR’s) were observed for 4 of 32 solid tumors (two neuroblastoma, one glioblastoma, and one rhabdomyosarcoma) and one of four ALL xenografts (48). Volasertib is a dihydropteridinone (Bl 6727) that targets the Polo-like kinase (Plk) family of proteins in an ATP-competitive manner at low nanomolar concentrations and thereby induces mitotic arrest and apoptosis (74). Plk1 is a serine/threonine-specific kinase that regulates multiple steps in mitosis and that is essential for progression through mitosis (75). Numerous lines of evidence suggest that Plk1 is oncogenic through driving cell cycle progression, and overexpression of the gene transforms NIH 3T3 cells (76). Plk1 is highly expressed in multiple cancers (75, 77, 78), and in some malignancies expression of Plk1 may be prognostic (77). Plk1 is over-expressed in several childhood cancers and cell lines. RNA interference and small molecule inhibitor screens suggest that Plk1 may be a relevant therapeutic target in a variety of pediatric malignancies including neuroblastoma, rhabdomyosarcoma, and OS (79–81).

From the *in vitro* sensitivity dataset, the elastic net regression algorithm selected 121 mRNA variables, 17 of which were lincRNAs, based upon the log rIC_50_ of 22 PPTP cell lines. The *in vitro* linear model with these 121 mRNA inputs predicted 36 xenograft outcomes (28 PD, 1 SD, 1 PR, and 6 CR) quite well with an area under the curve of 0.79. According to IPA, PKMYT1, DNHD1, KAT7, DDX39B, RASGRF1, and MAD2L1 biomarkers reportedly have interactions with the drug target, PLK1. Specifically, KAT7 (82), DNHD1 (83), DDX39B (84), and RASGRF1 (85) are known to have protein–protein interactions with PLK1 while mutant PLK1 (51–356 AA deletion) increases MAD2L1 protein localization to kinetochores from misguided chromosomes of metaphase cells (86) and PLK1 protein increases inhibition of active PKMYT1 (87) as well as increase phosphorylation of a PKMYT1 protein fragment (88). MAD2L1 and PKMYT1, both negatively correlated with rIC_50_, may point to PLK1 targets over-expressed when PLK1 is mutated. Interestingly, PKMYT1 is a protein kinase that plays an important role in mitosis by decreasing activation of CDK1 (89, 90) while increasing phosphorylation of CDK1 (89–91). The elevated PKMYT1 mRNA in sensitive cells is possibly indicating a cellular compensation for over active mitotic phase of the cell cycle due to mutated PLK1, and hence, these cell populations are ideal targets for PLK1 inhibition by BI6727. Furthermore, the good validation performance and significant relevance of discovered biomarkers prioritize this signature for additional validation. Recently, PLK1 was reported to phosphorylate PAX3-FOXO1 in alveolar rhabdomyosarcoma, and inhibition triggered tumor regressions (92).

### Glembatumumab vedotin

Glembatumumab vedotin is an antibody-drug conjugate (ADC) that combines an anti-GPNMB antibody with the anti-mitotic agent monomethyl auristatin E (vedotin) (93). When internalized, vedotin is released and results in cell cycle arrest and cell death (94). Glembatumumab vedotin showed *in vitro* cytotoxicity that was related to GPNMB expression, and it induced complete regressions in GPNMB-expressing melanoma and breast cancer xenografts (93, 95, 96).

The transmembrane glycoprotein NMB (GPNMB or osteoactivin), is primarily expressed in intracellular compartments (e.g., lysosomes and melanosomes) in non-malignant cell such as melanocytes, osteoclasts, and osteoblasts (97–99). GPNMB is also expressed on monocytes and dendritic cells, and its expression on the latter has been proposed to play a role in the inhibition of T-cell activation by antigen-presenting cells (APC) (100–102). Membrane GPNMP is over-expressed in hepatocellular carcinoma (103), breast cancer (95, 104), glioblastoma (105), and melanoma (93, 98), making it a reasonable candidate for targeted therapeutics. As shown in Figure 4, GPNMB is expressed highly in several OS xenografts [and also in one alveolar soft part sarcoma (ASPS) examined]. In a limited screen using models with high-level expression glembatumumab vedotin demonstrated intermediate to high activity in five of six OS xenografts, with a maintained complete response in three of the lines (52). In each of the lines that demonstrated a maintained complete response to glembatumumab vedotin (OS-2, OS-17, and OS-33), there is 2^+^ to 3^+^ staining for GPNMB by immunohistochemistry, although the percentage of cells positive is as low as 5% of tumor cells for one line. These observations support the position that while GPNMB expression may be necessary for tumor regression to glembatumumab vedotin treatment, it is not sufficient for response to this agent (52). The value of the expression data is further emphasized by searching publically available databases. For example, the single ASPS xenograft model expressed very high levels of GPNMB. Reference to limited patient data available, confirms high-level expression in all samples, suggesting that GPNMB-directed therapy may be valuable. However, it is recognized that ASPS is a slow-growing indolent tumor (as is the xenograft), hence whether an anti-mitotic “warhead” on glembatumumab would be effective would have to be explored in preclinical models.

### Seneca valley virus (NTX-010)

One of the agents evaluated through the PPTP was the replication competent picornavirus, Seneca Valley Virus (NTX-010) (106). NTX-010 is a newly discovered, naturally occurring picornavirus being developed as an oncolytic virus for human cancers. In a cell line screen of NTX-010, approximately half of cancer cells with one or more neuroendocrine properties were permissive and allowed selective infection (107). Notably, the most sensitive cell line, IMR-32, was derived from a childhood neuroblastoma. By contrast, only 3 of 80 non-endocrine cells were permissive to virus replication. The majority of non-permissive cancer cell lines do not bind and/or internalize NTX-010, suggesting that binding and entry through a productive internalization pathway is the primary determinant of viral tropism for neuroendocrine tumor cells. Neuroblastoma, Ewing sarcoma, as well as medulloblastoma and alveolar rhabdomyosarcoma demonstrate neuroendocrine markers. *In vitro* NTX-010 demonstrated a marked cytotoxic effect in a subset of the cell lines from the neuroblastoma, Ewing sarcoma, and rhabdomyosarcoma panels. *In vivo* the most consistent activity was observed for the rhabdomyosarcoma and the neuroblastoma panels, with all four of the alveolar rhabdomyosarcoma xenografts and four of five neuroblastoma xenografts achieving CR or maintained CR (106).

An overlooked aspect of our analytical approach is normality of rIC_50_. As mentioned previously, linear correlation and regression methods require that the response variable, rIC_50_, be normally distributed. NTX-010 is the only agent considered herein that exhibits a non-normal rIC_50_ profile. On a natural scale, the rIC_50_ profile appears to be discrete while on a logarithmic scale we observe normality for sensitive lines, i.e., any cell growth inhibition within dose range, whereas resistant lines, i.e., no inhibition at maximum dose, are saturated at the highest dose tested. Furthermore, measures of linear correlation in this context are likely highlighting differential sensitivity within sensitive population but are likely informative nonetheless.

The elastic net regression algorithm selected only 29 mRNA variables, two being lincRNAs, based upon the log rIC_50_ of 22 PPTP cell lines. The *in vitro* linear model did well when discriminating 22 xenograft outcomes (10 PD, 2 PR, 4 CR, and 6 MCR) given an area under the curve of 0.71. A notable mRNA feature is IFIH1 or interferon induced with helicase domain 1. IFIH1 is a picornavirus surveillance protein in innate antiviral response (108, 109). We speculate that a low level of IFIH1 is a marker of permissive replication in tumor cells. Taken together, high-level expression of CD56 (NCAM1) and low expression of IFIH1 accurately identifies 24 of 26 cell lines and xenografts as being sensitive to NTX-010 (106), as shown in the boxed area of Figure 5.

We further interrogated both *in vitro* and *in vivo* data to determine if other IFIH1-like factors are associated with sensitivity. A genome-wide unpaired *t*-test assuming that unequal population variances was computed between responders and non-responders where responders were sensitive cells or xenografts with maintained complete response (25) and non-responders were resistant cells or xenograft with progressive disease 1 (PD1); multiple hypothesis testing was corrected by Storey *q*-value (110) and all computational analyses were performed with Matlab Bioinformatics and Statistics toolboxes. As biologists and also from a practical statistical perspective, we search to see if discovered gene changes are enriched in a meaningful biological category. The hypergeometric probability distribution is appropriate to calculate the chance of observing category overlap at random and is utilized in, for example, the Broad Institute Molecular Signature Database (111). An insightful method to then prioritize categories is to integrate domain knowledge by scoring sets according to gene change consistency with literature findings and is heavily utilized in, for example, IPA.

Overall, we detected 692 Agilent mRNA variables when controlling a false discovery rate of 5%, i.e., Storey *q*-value <0.05. From IPA, we were able to infer by right-tailed Fisher’s exact test that discovered differential mRNA is predictive of several interesting functional categories related to virus attenuation as well as detecting highly elevated NCAM1, a receptor already speculated to be involved in NTX-010 cell entry (106). Notable categories of decreased activity in responders are infiltration by APC, antiviral response of cells, natural killer (NK) cell homeostasis, and activation of NK cells while a notable category of increased activity in responders is viral replication (vesicular stomatitis virus, replication of RNA virus, Murine herpesvirus 4).

The landscape of gene–gene correlations genome-wide that exists naturally either due to evolutionary redundancy or other factors is problematic when searching for mRNA correlates that are global and not confined to whatever cell lines happen to be in the training set. Interestingly, a NTX-010 lincRNA correlate (chr1:213453777–213480277; hg19) was upstream of RPS6KC1 and a gene–gene mRNA correlation was significant between these two. This observation points to the inherit difficulty of modeling basal mRNA and drug response. In this particular example, we can infer from genomic proximity that this non-coding mRNA feature is likely acting as a promoter of RPS6KC1. RPS6KC1, a candidate oncogene in endometrial cancer (112), is a meaningful drug–gene correlation given observations that NTX-010 tends to show response in neuroendocrine tumors (113). By establishing this “link” we were drawn to a significant drug–gene correlation that was de-prioritized by the elastic net regression algorithm. However, for the vast majority of proteins that are modified epigenetically or in distant trans interactions, such direct hypotheses are not easily formulated.

## Bioinformatics Tool Development and Availability

As new cancer genomic datasets come online, there is a need to rapidly develop tools, portal interfaces, and standards of analysis that robustly turn multiple sourced molecular data into an insightful axis of molecular relationships. The basic cancer dataset is a matrix of samples and genes with entries corresponding to a molecular readout such as gene expression or DNA copy number. A standard set of statistical methods adopted in the bioinformatics community for analyzing such a matrix are hierarchical cluster analysis (114), gene set enrichment analysis (115), sample randomization statistics (114–116), regression analysis (41, 42, 44), and dimensionality reduction methods (117–119). Additionally, most software tools for analyzing cancer genomic data (120–123) are made publicly available at no cost to non-profits with the caveat that there is no free lunch; prospective users typically agree to terms of conditions that include limited liability on the part of the tool creator.

## Critical Evaluation of Bioinformatics Analysis of PPTP Data

The obvious limitation of the bioinformatics analyses presented here is the relatively small sample size used to identify correlates. We have derived sensitivity data and, based upon expression profile differences between cell lines, have attempted to predict sensitivity to drugs of xenograft models. *In vitro*, cell lines from different tumor types (including leukemias) have been used, thus potentially biasing analyses to profiles exhibited by leukemia cells that tend to be more sensitive to many of the agents tested. To make correlations between *in vitro* sensitivity and *in vivo* models, we have used only the solid and brain tumors, and have excluded the leukemia models, as these have very different expression profiles (8). Thus, it is likely that analyses may be biased when there is a preponderance of one type of tumor in the sensitive or resistant cohort. Additional weaknesses include a failure to integrate exome mutation analysis, and changes in expression profiles subsequent to drug treatment (i.e., dynamic profiling). Despite these obvious weaknesses, the analyses do focus on specific genes/pathways that can be tested prospectively.

## Future Directions

Within the PPTP consortium, approximately 150 patient-derived xenograft models have been established. Most have been characterized by expression profiling and exome sequencing, hence a valuable omics database has been created against which new agents can be profiled. However, it is clear that to accurately represent molecular subtypes of different cancers additional models need to be established. Several novel agents identified in the PPTP screen are in phase I/II testing for treatment of childhood cancer. For sarcomas, the models identify some anti-mitotic agents as being highly active. Whether this reflects an increased rate of proliferation in models compared to patient tumor, or is revealing the Achilles Heel of these cancers, is open to debate. The activity of signaling inhibitors against the xenograft models has been somewhat disappointing, but this may reflect the lack of activity in human cancers overall. Certainly, in models with “actionable” mutations, specific inhibitors show impressive activity. However, it is clear that development of this type of targeted therapeutic must differ from the paradigm used for developing cytotoxic agents.

As was mentioned previously, the real power of cancer genomics data lies in the ability to integrate multiple molecular data sources. Open web portals that provide access to publicly available multi-source cancer genomic data, largely from the Tumor Cancer Genome Atlas (TCGA), are advancing our understanding of cancer genomes (124) and their susceptibility to anti-cancer agents. Literally within a click or two an investigator can begin to hypothesize how their gene of interest or empirical pathway is active in specific cancer patient populations or associated with cancer cell drug sensitivity or resistance. Here, we have discussed the value and limitations of deriving relationships between *in vitro* cell line sensitivity and *in vivo* responsiveness to several agents. Potentially, identification of synergistic combinations *in vitro* can be tested in xenograft models to develop rational combination therapies. The examples were chosen to illustrate the value and limitations of this approach. Further refinement and validation of such “signatures” are required, possibly using a further test set of xenografts, or through modulation of genes by RNA interference approaches. Ultimately, it will be important to determine whether such approaches are relevant to patient responses to single agents or to complex therapeutic regimens.

## Conflict of Interest Statement

The authors declare that the research was conducted in the absence of any commercial or financial relationships that could be construed as a potential conflict of interest.
